# Creative entrepreneurship as a socially embedded form of work: a sociological ecology for the platform society

**DOI:** 10.3389/fsoc.2026.1959266

**Published:** 2026-10-02

**Authors:** Linchu Rajan, Lekshmi Mohan, Joel George, Ajimon George, Anoop Tom Thomas, Maria Susan Mathew

**Affiliations:** 1 St. Berchmans College, Changanassery, Kerala, India; 2 Kalasalingam Academy of Research and Education (Deemed to be University), Krishnan Kovil, India; 3 Newman College, Thodupuzha, India; 4 Marian College Kuttikkanam Autonomous, Idukki, Kerala, India; 5 St. Dominic's College, Kanjirapally, Kerala, India

**Keywords:** creative entrepreneurship, creative work, digital labor, identity construction, platform governance, platform society, relational capital, sociology of work

## Abstract

Creative entrepreneurship increasingly combines creative production with entrepreneurial, communicative, and platform-mediated work. Existing scholarship has generated important insights into opportunity recognition, innovation, value creation, and creative labor, but these perspectives often examine entrepreneurial activity, social relations, cultural recognition, and platform governance as analytically separate processes. This Perspective develops the Sociological Ecology of Creative Entrepreneurship (SECE), a conceptual framework that positions creative entrepreneurship as socially embedded work within platform society. Drawing on economic sociology, sociology of work, labor process theory, digital sociology, and platform studies, SECE integrates five analytically distinct but relationally connected dimensions: identity construction, relational capital, platform governance, cultural legitimacy, and institutional embeddedness. The framework is presented as a relational and multilevel heuristic rather than a tested causal model. It proposes that creative entrepreneurial practice emerges through interactions among workers, audiences, collaborators, platforms, cultural fields, and institutions. The Perspective further considers how these relations are shaped by power, inequality, and human–AI interaction and identifies directions for empirical research into platform-mediated creative work, occupational sustainability, and changing forms of employment.

## 1 Introduction

The nature of work has changed significantly over the past two decades. The rapid growth of digital platforms, social networks, artificial intelligence (AI), and creator economies has generated new forms of work whose boundaries are often difficult to define within traditional organizational structures. People are no longer exclusively engaged in one occupation, or engaged in self-employment, or engaged in a certain business, but they are more and more likely to combine multiple occupations, to work in a world of flexible, uncertain and continuously changing labor markets that are mediated by the Internet (Casas-Cortés et al., 2023; van Dijck et al., 2018). Creative entrepreneurship represents an emerging mode of work characteristic of the platform society.

Creative workers—including artists, musicians, designers, writers, photographers, filmmakers, illustrators, performers, craftspeople, and digital content creators— increasingly occupy hybrid occupational roles. Their professional activities are not limited to creative production but also involve branding, audience engagement, digital marketing, community management, collaboration, platform optimization, and entrepreneurial decision-making. Thus, creative entrepreneurship is not simply about the commercialization of artistic products but involves a form of work embedded in digital, cultural, and economic networks (Hesmondhalgh, 2013; Howkins, 2002).

Existing scholarship has made a significant contribution to understanding creative entrepreneurship from entrepreneurship, commerce and management perspectives. The emphasis of the research has been placed on the following factors as key influences on the success of an entrepreneur: entrepreneurial orientation, opportunity recognition, innovation capability, resource mobilization, business model development, and value creation (Bridgstock, 2013; Fillis, 2010; Shane and Venkataraman, 2007). In the same way, research on the creative economy has shown great interest in the creative industry's importance for innovation, jobs, and regional development (Caves, 2003; Hesmondhalgh, 2013). Together these viewpoints have created a distinctive area of study for entrepreneurship and business in terms of creative entrepreneurship.

But the platformization of creative labor calls into question theoretical issues that go beyond the traditional models of entrepreneurship. Increasingly, entrepreneurial success is tied not just to business skills, but to social visibility, digital reputation, audience engagement, **algorithmic** endorsement systems, and institutional legitimacy. Creative entrepreneurs are constantly negotiating their professional identities in the context of audience, fellow professionals, technological systems, and the platform governance systems that shape access to visibility and economic opportunity (Gillespie, 2019; van Dijck et al., 2018). These developments highlight that creative entrepreneurship is essentially socially embedded and, therefore, requires stronger engagement with sociological theory.

Scholarly work has been done recently that provides significant bases for this argument. Empirical research suggests that social media provides an entrepreneurial environment that can be used strategically to help the artrepreneurs build audiences, professional legitimacy and entrepreneurial opportunities (George and Mathew, 2025). The recent research on AI and creative practice and digital strategic engagement underscores how digital technologies have become more integral to creative production, communication, collaboration and entrepreneurial decision-making (George and Susan Mathew, 2025; Susan Mathew et al., 2026). Concurrently, phenomenological studies uncover social costs of platform dependence, such as the visibility labor, identity fragmentation, temporal compression and reputational vulnerability (Susan Mathew and George, 2026). These studies indicate that digital creative entrepreneurship creates opportunities while also introducing new pressures and forms of dependence.

Existing scholarship therefore provides important but partial explanations of creative entrepreneurship under platform conditions. Research on entrepreneurial behavior emphasizes opportunity recognition, innovation, and value creation, while sociology of work draws attention to occupational identities, social relations, autonomy, insecurity, and the organization of labor. Platform studies further demonstrate that digital infrastructures shape visibility, participation, and cultural production, whereas cultural and institutional perspectives explain how recognition and resources are socially conditioned. These perspectives are complementary, but they are not always integrated into a common account of how creative entrepreneurship is organized as work within platform-mediated environments.

This Perspective addresses that conceptual gap by proposing the Sociological Ecology of Creative Entrepreneurship (SECE). The role of SECE is not to assert that the concepts of identity construction, relational capital, platform governance, cultural legitimacy and institutional embeddedness are new in themselves. Instead, it serves as a way to combine these well-known sociological concepts in a relational perspective of creative entrepreneurship as socially embedded work. SECE thus redirects the analytical attention from the individual capabilities of the entrepreneur and toward the relations that are the way in which creative work is organized, recognized, enabled, constrained, and sustained.

SECE is not a predictive or empirically proven causal model, but rather a multilevel and relational heuristic model. It is situated at the intersection of the individual occupational identity, interpersonal and network relations, platform infrastructures, cultural fields, and institutional environments. Its explanatory value lies in providing a “relational vocabulary” for examining the dimensions interact in shaping the organization of creative entrepreneurial practice. For instance, the level of cultural legitimacy may be influenced by relational resources, and platform governance may influence the extent to which relational resources are translated into visibility and opportunity. This conversion can also be further facilitated or limited by institutional support. The framework thus offers an integrated sociological perspective that can be used to analyze creative entrepreneurship as a form of work in social, technological, cultural and institutional relations.

This Perspective has three major contributions. First, it situates creative entrepreneurship within the sociology of work by conceptualizing it as occupational practice rather than solely as entrepreneurial behavior. Second, it brings together perspectives from economic sociology, digital sociology, labor process theory and platform studies within the SECE framework. Third, it establishes a research agenda to explore the impact of social relations, platform infrastructures, cultural recognition, institutional conditions, power and inequality on creative entrepreneurial activities in platform society.

## 2 Conceptual gap and theoretical contribution

The conceptual gap addressed by SECE concerns the fragmented treatment of the social conditions under which creative entrepreneurship is organized as work. Entrepreneurship scholarship has traditionally emphasized opportunity recognition, innovation, commercialization and value creation, while research on creative labor has highlighted the cultural and occupational conditions surrounding creative production. Platform studies have, in turn, shown how digital infrastructures influence visibility, participation and monetization. These lenses do not offer a holistic explanation; however, they provide complementary insights into how identity, relationships, platform governance, cultural recognition and institutional context work together in shaping creative entrepreneurial practice.

To address this gap, SECE views creative entrepreneurship as an occupation that is socially embedded and exists in a platform-mediated ecology. It is not additive, but rather integrative: identity construction enables us to understand how creative workers construct their identities and how they negotiate who they are as a creative worker; relational capital explains how social resources are embedded in opportunities, support and recognition; platform governance provides a look at the infrastructural condition that enables visibility and participation; cultural legitimacy helps us to understand processes of recognition and valuation; and institutional embeddedness situates creative entrepreneurship within wider systems of education, policy, funding, regulation and cultural institutions.

The five dimensions are distinguishable analytically and connected relationally. SECE does not argue that the entrepreneurial outcomes are determined by any single dimension alone. Instead it emphasizes how the dimensions can reinforce, constrain, and/or transform each other in specific platform and institutional settings. For instance, relational resources can be used to gain visibility and recognition, and platform governance can shape the convertibility of the resource to audience access and/or economic opportunity. In the same way, institutional support can enhance the resources of creative entrepreneurs, as well as the level of legitimacy associated with creative work forms.

As a consequence, SECE should be conceptualized as a relational, multilevel framework that brings together and synthesizes the available sociological literature for a relational and multilevel understanding of platform-based creative labor. It is about providing explanations for creative entrepreneurship that are not only economic but also social, cultural, institutional, and relational, in the sense that the activity is an act of work, which occurs in the process of a relationship between workers, audiences, collaborators, platforms, cultural fields and institutions. The framework is suggested as a potential basis for empirical studies in the future, not as an established causal model.

### 2.1 Unit of analysis and boundary conditions

SECE primarily focuses on the occupational practice of the creative entrepreneur as its unit of analysis, acknowledging that this practice is embedded in relations that span the individual, network, platform, cultural and institutional level. The framework is designed for creative entrepreneurs for whom access to audiences, reputation, market opportunities or income is significantly dependent on digital platforms. It is not therefore suggested as a model of all creative work, or as a model of all entrepreneurship. Its relevance may vary depending on the creative sector, platform type, degree of platform dependency, geographical context, and regulatory environment, as well as workers' access to social, cultural, economic, and technological resources. This boundary distinguishes creative entrepreneurship from creator work, freelance cultural work, and platform labor more broadly: SECE focuses specifically on entrepreneurial creative practice in which creative production is combined with responsibility for market access, audience development, professional positioning, or value capture.

## 3 Why creative entrepreneurship requires a sociological perspective

Creative entrepreneurship has been traditionally conceptualized as an action that involves opportunity recognition, innovation, commercialization and value creation (Caves, 2003; Shane and Venkataraman, 2007). These viewpoints have led to valuable insights into entrepreneurial behavior and the business process for commercializing creative products. Regardless, they are still mostly market-oriented, creating a vision of the creative entrepreneur as a rational economic player in whose success is largely due to entrepreneurial capacity and business performance. This perspective is informative, but it captures only part of the organization of contemporary creative work.

### 3.1 Creative entrepreneurship as platform-mediated work

The digitization of creative industries has altered not only how creative products are distributed but also how creative work is organized. Digital platforms increasingly operate as infrastructures through which production, distribution, audience access, evaluation, and monetization are coordinated (Poell et al., 2021; van Dijck et al., 2018). For creative entrepreneurs, this means that entrepreneurial activity extends beyond producing creative outputs to include maintaining visibility, developing audiences, managing digital identities, monitoring platform metrics, responding to audience expectations, and adapting practices to changing platform conditions. Creative work consequently involves an expanding combination of artistic, entrepreneurial, communicative, and relational activities (Duffy, 2017; Hesmondhalgh and Baker, 2011).

From a sociological point of view, these changes make it increasingly difficult to distinguish between employment, self-employment, entrepreneurship and independent creative activity. Creative entrepreneurs may operate as self-employed or independent workers, but still rely on infrastructures that shape their audience access, visibility and income. The study of digital labor indicates that platform work can be flexible and autonomous and also involve forms of algorithmic control, insecurity and unequal bargaining power (Hjorth and Lehdonvirta, 2017; Wood et al., 2019). Likewise, studies on creative labor reveal the conflicting and contradictory nature of autonomy and self-realization, commercial requirements, emotional employment and self-exploitation (Duffy, 2017; Hesmondhalgh and Baker, 2011).

Platforms can use ranking systems, recommendation mechanisms, metrics, monetization rules, content policies, and terms of service as means of structuring work. This is important from a labor-process perspective because control can be embedded in platform infrastructures and enacted through data-driven evaluation (Kellogg et al., 2019) without the worker having the formal independence of an employer.

Algorithmic management research has shown that digital systems can direct, evaluate and discipline work in a variety of ways including recommendation, recording, ratings, rewarding and replacement (Kellogg et al., 2019). In a creative context, there is no clear causal connection between such mechanisms and the worker's behavior, but they can affect when, how, and how often the worker produces, as well as how they are perceived. A tension between autonomy and dependence at the platform level is then seen as a feature of platform-mediated creative entrepreneurship (Cotter, 2019; Wood et al., 2019).

This tension is the key element of the sociological interpretation that follows. Creative entrepreneurs are located on the nexus of several occupational domains—requiring them to create a product, make decisions as entrepreneurs, interact with an audience, present the product, and adapt the platform. These activities constitute neither conventional entrepreneurship nor conventional employment. Rather, it is a form of work distributed across multiple organizational arrangements, involving individuals, social networks, platform infrastructures, cultural communities, and institutional environments.

Empirical and conceptual evidence have emerged in recent years that can facilitate the understanding of creative entrepreneurship as socially organized and platform mediated work. Studies of social media entrepreneurship suggest that digital platforms have the potential to serve as strategic spaces for creative entrepreneurs to build their audiences, professional profiles, networks and entrepreneurial opportunities (George and Mathew, 2025). Creative entrepreneurship is also found to be communicative and relational and is associated with practices that go beyond the use of technologies (Susan Mathew et al., 2026). But, in addition, there are also aspects of unpaid and precarious work. The studies of creative and social media labor have focused on the concept of aspirational labor, gender disparities and the challenges of earning a sustainable income from creative activity (Duffy, 2015; Duffy et al., 2021). The study of digital labor also reveals conflicts between being flexible and free from supervision on the one hand and being controlled by algorithms, being trapped in unequal bargaining positions, and experiencing insecurity over their livelihoods on the other (Hjorth and Lehdonvirta, 2017; Wood et al., 2019).

The visibility of the platform is therefore more than just a technical result. Algorithmic systems are interpreted, adapted and transformed into forms of practical knowledge by influencers and other algorithm-dependent workers, suggesting that algorithms can shape, but do not necessarily dictate, behavior (Cotter, 2019). Visibility labor can itself become an occupational necessity, involving continuous activities carried out to remain visible and relevant to the platform environment (Abidin, 2016). The dynamics contribute to a sociological understanding of creative entrepreneurship as a form of work that extends beyond creative production that involves identity management, audience maintenance, platform adaptation and reputational work.

This is further complicated by AI. SECE does not conceptualize AI as a substitute for human creativity but rather focuses on human–AI interaction as mediated by organizational decisions, access to technological infrastructures, and institutional arrangements. AI can transform creative work, decision-making, and authorship, as well as the skill sets needed to do creative work and the professional identity of the creative worker, and may also lead to concerns around ownership, legitimacy and occupational autonomy (Jarrahi, 2018). Rather than being about the technology itself, the value of AI is defined by the context in which it is used in creative practice.

Combined, this literature indicates that entrepreneurial capacity is not the only component which can explain creative entrepreneurship. Creative workers are situated in networks of people, culture, platforms, institutions, and all can provide opportunities as well as produce constraints. SECE thus coalesces these circumstances to explore creative entrepreneurship as a socially situated practice of work.

## 4 Conceptual development of the SECE framework

The five dimensions of SECE were derived through conceptual integration rather than statistical factor extraction or *post hoc* thematic grouping. Three requirements guided the selection of these dimensions: the first is that each should symbolize a different sociological context for organizing, or experiencing, creative activity; the second is that each should have a solid theoretical basis; the third is that each should refer to some aspect of organizational and/or experiential platformism that cannot be fully explained by the individualistic perspective on entrepreneurial capability. Identity construction is about the negotiated process and construction of occupational identity, relational capital is about social resources embedded in networks and communities, platform governance is about the infrastructural organization of visibility, participation and monetization; cultural legitimacy is about the processes of social recognition and valuation; and institutional embeddedness refers to positioning creations in the fields of education, policy, funding, regulation and cultural institutions. The dimensions are analytically distinct but relationally connected, so that SECE can investigate the interactions between different types of embeddedness without having to start with a particular cause and proceed in a definite order.

The five-domain structure therefore provides an integrated vocabulary for analyzing creative entrepreneurship across individual, relational, technological, cultural, and institutional levels.

## 5 The sociological ecology of creative entrepreneurship (SECE)

The Sociological Ecology of Creative Entrepreneurship (SECE) is a conceptual framework that defines creative entrepreneurship as socially situated work that is formed in the relation between people, technologies, institutions and cultural systems. The framework consists of the five analytically distinct but relationally connected dimensions of identity construction, relational capital, platform governance, cultural legitimacy and institutional embeddedness (Table 1 and Figure 1). These dimensions represent conditions through which creative work is organized, recognized, enabled, and constrained rather than independent causal variables.

**Table 1:** Core dimensions and sociological foundations of the SECE framework.

SECE dimension	Conceptual focus	Theoretical foundation	Role within the ecology
Identity construction	Negotiation of artistic and professional identity	Identity construction and occupational identity (Ashforth and Schinoff, 2016)	Shapes how creative workers position and present themselves within social and occupational environments
Relational capital	Networks, audiences, collaborators, and social resources	Embeddedness and social network theory (M. Granovetter, 2008; M. S. Granovetter, 1973)	Provides relational resources through which information, recognition, collaboration, and opportunities may be accessed
Platform governance	Algorithmic coordination, visibility, metrics, monetization, and platform rules	Platform studies and labor-process perspectives (Kellogg et al., 2019; van Dijck et al., 2018)	Structures conditions of participation, visibility, evaluation, and platform dependence
Cultural Legitimacy	Recognition, reputation, authenticity, and cultural valuation	Cultural capital and legitimacy theory (Bourdieu, 2008; Suchman, 1995)	Shapes how creative work and professional identities are socially recognized and valued
Institutional embeddedness	Education, policy, funding, cultural institutions, and occupational infrastructures	Economic sociology and institutional perspectives	Conditions access to resources, protection, recognition, and occupational sustainability

**Figure 1:**
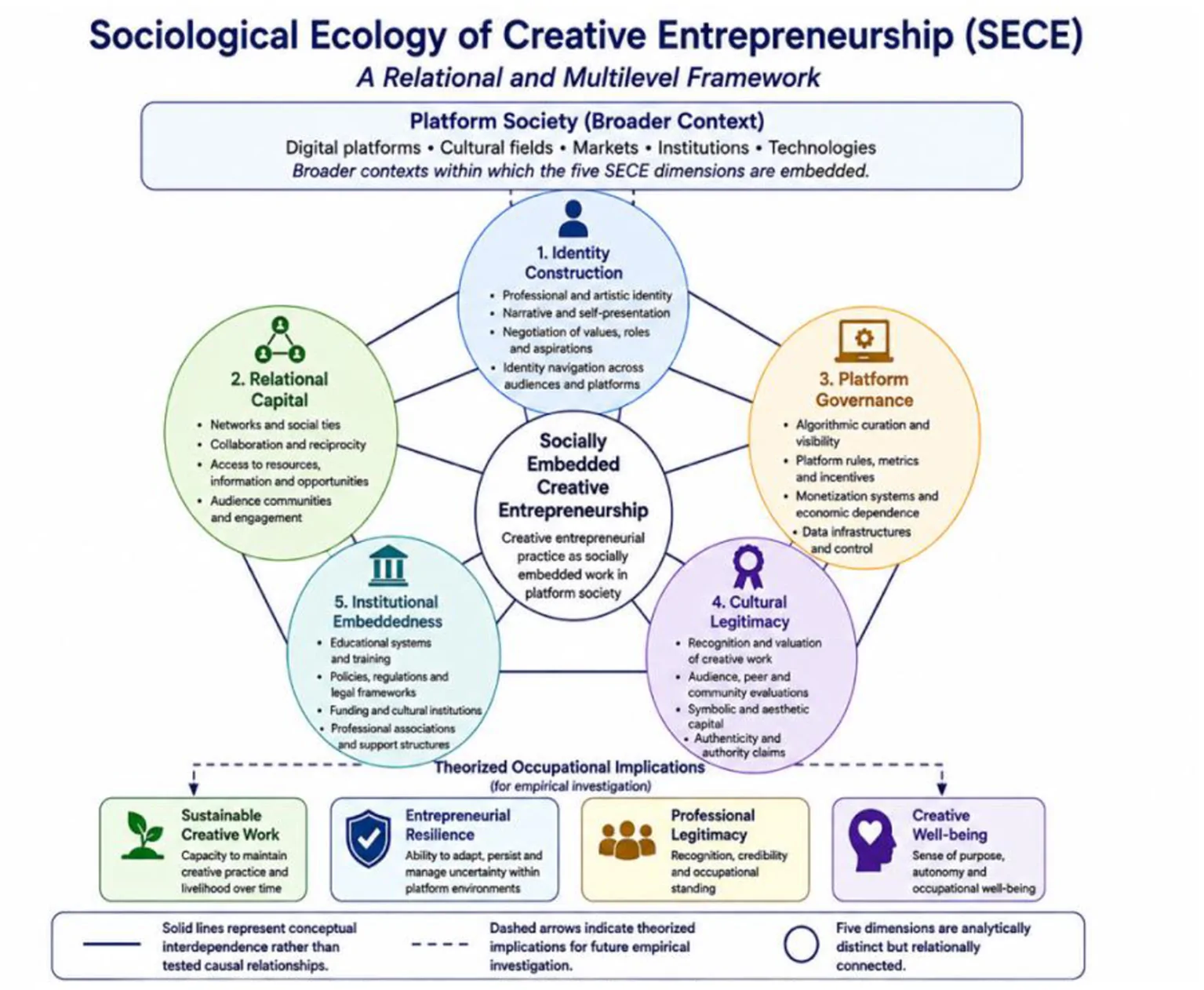
Sociological ecology of creative entrepreneurship (SECE). The framework conceptualizes creative entrepreneurship as socially embedded work arising through the relational interaction of identity construction, relational capital, platform governance, cultural legitimacy, and institutional embeddedness. Connecting lines represent conceptual interdependence rather than tested causal relationships. The four downstream elements represent theorized occupational implications for future empirical investigation and are not presented as empirically established effects of SECE.

### 5.1 Identity construction

Entrepreneurs as creatives continuously negotiate and build a professional identity during engagement with audiences, colleagues, co-creators and platforms. Identity construction thus extends beyond just personal branding to involve the negotiation of artistic values, entrepreneurial goals, audience expectations and platform visibility. Drawing on scholarship on identity construction, professional identities can be understood as dynamic constructions formed through social and organizational interactions (Ashforth and Schinoff, 2016). Platform-mediated creative production can see creatives reveal their true artistic persona whilst adjusting communication and content to platform conventions. This raises the prospect of tension between artistic independence, expectations of business and visibility. SECE views identity construction as an active, rather than static, occupational process rather than an established personal characteristic.

### 5.2 Relational capital

Creative entrepreneurial activity is rooted in relationships with audiences, collaborators and peers, professional networks, communities and other intermediaries. Relational capital refers to the social resources embedded in these relationships, which can facilitate information sharing, collaboration, recognition, trust, and access to opportunities. Embeddedness and social networks highlight the interconnectedness between economic behavior and social relations (M. S. Granovetter, 1973). These relationships can happen across geographically distributed networks in a digital environment and involve audience communities, online collaborators, professional peers, and platform-mediated relationships. SECE therefore sees relational capital not only as a promotional tool but as a state wherein creative work becomes socially connected and/or sustainable.

### 5.3 Platform governance

Platform governance refers to the rules, infrastructures, algorithmic systems, metrics, and organizational arrangements that govern participation and access to audiences on platforms. Platforms are therefore not merely neutral spaces for creative activity; they also participate in cultural production by shaping distribution, visibility, monetization, and interaction (Poell et al., 2021; van Dijck et al., 2018). From a labor-process perspective, platform governance can introduce forms of control without conventional managerial hierarchy. Algorithmic systems can suggest, rank, record, rate, reward, or bar activities, which can influence the context in which creative workers arrange activities (Kellogg et al., 2019). Research on the digital influencers also suggests that they read and react to their perceived algorithmic expectations, indicating that algorithms shape behavior, but do not completely control it (Cotter, 2019). In SECE, platform governance is therefore framed as a form of algorithmic coordination and infrastructural dependency rather than as a deterministic force.

### 5.4 Cultural legitimacy

Creative activity involves not only the production and exchange of creative products but also processes of recognition and valuation. Cultural legitimacy is defined as the degree of credibility, appropriateness, value or authenticity attributed to creative work, identities and practices by relevant audiences, peers, institutions and cultural communities. The concept of cultural capital provides an explanation for the social distribution of recognition and status, while legitimacy theory emphasizes the role of audience evaluations in establishing and maintaining legitimacy (Bourdieu, 2008; Suchman, 1995). Cultural legitimacy can be negotiated in platform environments, by means of recognition from peers, audience reactions, institutional support, and platform metrics. The sources of recognition can also be independent: recognition can be gained through visibility, but not necessarily through professional legitimacy, and practices that build algorithmic reach can have a negative impact on artistic values. In such a sense, cultural legitimacy is a process of social valuation through which recognition, reputation, authenticity and professionalism are negotiated.

### 5.5 Institutional embeddedness

Creative entrepreneurial activity takes place in institutional contexts such as education, cultural institutions, professional associations, public policy, funding systems, intellectual property systems and other systems of occupational infrastructure. These arrangements impact access to resources, recognition for their profession, protection, and opportunities for their career. The concept of institutional embeddedness thus goes beyond individual and platform-level encounters to examine how institutional contexts shape the sustainability of creative practices. These conditions vary across creative fields, regions, and regulatory settings, contributing to differences in platform-mediated creative entrepreneurship experiences.

### 5.6 Stratified embeddedness, power and inequality

The ecology depicted in SECE is not “socially neutral”. Access to networks, cultural recognition, technological resources, platform visibility, and institutional support is unequally distributed. Creative workers therefore enter platform environments equipped with varying levels of economic, cultural, social and institutional resources. Social media creative labor research shows that inequalities that emerge on creative labor within the domain of traditional media have the potential to be reproduced within the social media environment (Duffy, 2015, 2017; Duffy et al., 2021). These may also differ regionally, linguistically, with respect to disability, the creative sector, type of platform and regulatory environment. SECE, therefore, does not consider embeddedness to be monolithic and universally applied but as a stratified and context-dependent process.

## 6 Implications for work, employment and organizations

SECE contributes to the sociology of work by conceptualizing creative entrepreneurship as a socially organized occupational practice rather than solely as an individual entrepreneurial activity. Contemporary creative workers frequently combine production, entrepreneurship, communication, audience management, self-presentation, and platform adaptation within the same occupational role. Creative entrepreneurship consequently occupies an ambiguous position between self-employment, independent creative work, entrepreneurial work, and platform-mediated labor.

The framework also extends the analysis of organizational control. Platform-mediated creative work opens a possibility to configure work in a new way, where the infrastructures, algorithms, metrics, monetization systems and platform rules can shape creative work without being based on an old managerial hierarchy. These forms of coordination can coexist with substantial worker autonomy, creating a tension between independence and infrastructural dependence (Kellogg et al., 2019; Wood et al., 2019). This configuration also shifts organizational power from direct managerial supervision toward infrastructural forms of coordination embedded in platform rules, metrics, and data-driven evaluation.

SECE also helps to inform discussions about the quality and sustainability of creative practice by collecting and assembling the social resources and constraints of occupational practice. Platform governance can shape and/or constrain visibility, cultural legitimacy can affect professional recognition, relational capital can enable collaboration and opportunity, and institutional embeddedness can offer and/or deny resources to occupational continuity. These relationships indicate the importance of examining individual capabilities in relation with the occupational outcome.

Finally, the framework emphasizes the stratification of embeddedness. Creative entrepreneurs differ in their access to networks, cultural capital, technological resources, institutional support, and platform visibility. Platform-mediated work may increase market access and opportunities for self-expression while also intensifying visibility labor, insecurity, and power imbalances experienced by workers (Duffy, 2017; Hjorth and Lehdonvirta, 2017; Wood et al., 2019). Power and inequality should then be seen as forces that influence the functioning of the five SECE dimensions and not as add-ons.

## 7 Future research agenda

SECE generates several directions for empirical investigation. First, longitudinal research could examine how identity construction changes as creative entrepreneurs move across different stages of platform dependence, professional development, and occupational transition. Second, qualitative and social-network research could investigate how relational capital contributes to cultural legitimacy and whether platform visibility strengthens or weakens the conversion of relational resources into professional recognition. Third, comparative platform research could examine how recommendation systems, monetization structures, moderation practices, visibility mechanisms, and creator policies shape occupational autonomy and dependency. Fourth, comparative institutional research could examine how education, cultural institutions, public policy, copyright regimes, and funding structures influence the sustainability of creative careers across sectors and countries. Fifth, intersectional research could investigate how gender, class, geography, language, disability, creative sector, and access to technological resources condition the operation of SECE's five dimensions. Sixth, digital ethnography, interviews, and platform trace-data research could examine how human–AI collaboration affects professional identity, creative autonomy, authorship, skills, and cultural legitimacy. Finally, mixed-methods studies could examine specific relationships among SECE dimensions and theorized occupational implications while complementing, rather than replacing, longitudinal, qualitative, comparative, and network-based approaches.

## 8 Conclusion

Creative entrepreneurship is increasingly organized through occupational arrangements in which creative production is combined with entrepreneurial activity, audience engagement, platform adaptation, and institutional negotiation. Existing entrepreneurial approaches provide important explanations of opportunity, innovation, and value creation, but they do not fully capture the social organization of creative work under platform conditions.

This Perspective proposed the Sociological Ecology of Creative Entrepreneurship (SECE) as an integrative sociological heuristic for addressing this limitation. SECE brings together five analytically distinct but relationally connected dimensions—identity construction, relational capital, platform governance, cultural legitimacy, and institutional embeddedness—to conceptualize creative entrepreneurship as socially embedded work. Its contribution lies not in presenting these dimensions as individually novel, but in theorizing their interaction within a common framework for analyzing platform-mediated creative occupations.

The framework also emphasizes that embeddedness is stratified and that creative workers experience platform environments differently according to their social, cultural, technological, and institutional resources. AI and other digital technologies are similarly understood as sociotechnical conditions whose implications depend on organizational and institutional arrangements rather than as autonomous determinants of creative work. SECE therefore offers a conceptual foundation for future research on changing occupational boundaries, platform-mediated labor, algorithmic coordination, professional identity, cultural legitimacy, and the sustainability of creative work.

## Data Availability

The original contributions presented in the study are included in the article/supplementary material, further inquiries can be directed to the corresponding author.
